# Effects of high-gravity acceleration forces and anti-gravity maneuver on the cardiac function of fighter pilots

**DOI:** 10.1038/s41598-024-59274-2

**Published:** 2024-04-16

**Authors:** Moon-Seung Soh, Jae-Hyuk Jang, Jin-Sun Park, Joon-Han Shin

**Affiliations:** 1 Department of Cardiology, Ajou University School of Medicine, Suwon, Korea; 2 Division of Cardiology, Uijeongbu St. Mary’s Hospital, The Catholic University of Korea, Uijeongbu, Korea

**Keywords:** Pilot, Echocardiography, Gravity acceleration forces, Valsalva maneuver, Environmental sciences, Space physics, Cardiology

## Abstract

The fighter pilots exposed to high gravitational (G) acceleration must perform anti-G maneuvers similar to the Valsalva maneuver. However, the effects of high-G acceleration and anti-G maneuvers on cardiac function have rarely been studied. This study aimed to investigate the effects of high-G forces on cardiac function of fighter pilots. Fighter pilots who underwent regular health check-ups and echocardiography were included (n = 29; 100% men, 41 ± 10 years old; mean flight time, 1821 ± 1186 h). Trainees who had not experienced any flights were included in the control group (n = 16; 100% men, 36 ± 17 years old). Echocardiographic data included left ventricular chamber size, systolic and diastolic functions, right ventricular systolic pressure (RVSP), inferior vena cava (IVC) collapsibility, and tricuspid annular plane systolic excursion (TAPSE). No significant differences in left ventricular ejection fraction, RVSP, or IVC collapsibility were observed between two groups. In the multivariate linear regression analysis with total flight time as an independent continuous variable for fighter pilots, TAPSE was positively correlated with total flight time. The experience of fighter pilots who were exposed to high-G acceleration forces and anti-G maneuvers did not cause cardiac structural changes, but the exposure might be associated with right heart function changes.

## Introduction

Environmental factors, such as hypoxia, high altitude, noise, and climate change, can affect the cardiovascular system^1–3^. Gravitational (G) force plays an essential role in the functioning of the cardiovascular system through physiological adaptation^4–6^. Changes in G acceleration, high altitude, and low air pressure have short- and long-term effects on a pilot’s cardiovascular function^1–6^. These factors are important and must be considered for jet fighter pilots exposed to high altitudes and harsh G accelerations during flights.

The current literature on jet pilots mostly comprises cross-sectional studies that focus on the prevalence of cardiovascular disease in these pilots or their differences from controls. A 2017 study comparing the risk of cardiovascular diseases in National Aeronautics and Space Administration astronauts and control participants reported no significant differences between the two groups with respect to diseases such as myocardial infarction, heart failure, and stroke^7^. Similarly, a study that analyzed ischemic heart disease and related risk factors in Hungarian air force pilots over a 10-year period reported no significant changes^8^. However, these studies only demonstrated the healthy worker effect and were limited to clarifying the direct effects of the high-intensity environment to which pilots are exposed.

Furthermore, attempts have been made to precisely investigate the cardiovascular effects and structural changes in pilots exposed to high-G acceleration. A 1996 study of Israeli pilots compared the echocardiography results of high-G acceleration-exposed pilots with those of controls; however, no significant differences were observed^9^. Moreover, a 2010 study comparing the echocardiographic results of pilots exposed to high-G acceleration and control pilots reported no clinical differences in heart size or function^10^. However, these studies were retrospective and targeted only categorized pilots, and the exposure period to G-acceleration was short.

Interestingly, jet fighter pilots usually perform an anti-G respiratory maneuver, which is physiologically similar to the Valsalva maneuver, to prevent fainting owing to changes in G acceleration during flight. This maneuver is performed by tightening the abdomen and taking short but regular breaths through the mouth instead of the nose. Similarly, the Valsalva maneuver is performed by exhaling forcefully against the closed glottis, which activates the autonomic nervous system and increases intrathoracic and intraabdominal pressures^11,12^. The anti-G respiratory maneuver is a form of positive-pressure ventilation that resembles the Valsalva maneuver in terms of venous return and cardiac physiology on the right side.

The effects of high-G acceleration and repetitive anti-G maneuvers on cardiac function and structure, including those of the right heart, remain unknown. Thus, this study aimed to investigate functional and structural cardiac changes using echocardiographic data of fighter pilots who continuously fly at high-G acceleration.

## Results

In total, 29 fighter pilots and 16 trainees were enrolled in the flight and non-flight groups, respectively. Average flight time was approximately 1821 ± 1186 h, and the average age was 41 ± 10 years in the flight group. There were no significant differences in age, height, medical history, family history, BP, or blood glucose levels between the flight and non-flight groups (Table 1). However, the flight group had higher body weight and total cholesterol levels than the non-flight group.

**Table 1:** Comparison of clinical demographics between the non-flight and flight groups.

	Characteristics	Non-flight group (n = 16)	Flight group (n = 29)	*P* value
Clinical characteristics	Male (n [%])	16 (100%)	29 (100%)	–
	Age (years)	36 ± 17	41 ± 10	0.239
	Total flight time (h)	–	1821 ± 1186	–
	Height (m)	1.74 ± 0.04	1.75 ± 0.04	0.293
	Weight (kg)	70.0 ± 8.0	76.9 ± 9.5	0.010
	Systolic BP (mmHg)	123 ± 9	125 ± 13	0.991
	Diastolic BP (mmHg)	81 ± 8	77 ± 8	0.103
History	Cardiovascular disease (n (%))	1 (6.2%)	1 (3.4%)	0.590
	Hypertension (n (%))	1 (6.2%)	3 (10.3%)	0.552
	Dyslipidaemia (n (%))	0 (0%)	3 (10.3%)	0.542
	Family history (n (%))	0 (0%)	1 (3%)	0.592
	Alcohol (n (%))	10 (63%)	23 (79%)	0.296
	Smoking (n (%))	4 (25%)	11 (38%)	0.514
Laboratory data	Total cholesterol (mg/dL)	164 ± 34	192 ± 49	0.009
	Low-density lipoprotein (mg/dL)	106 ± 26	125 ± 38	0.118
	High-density lipoprotein (mg/dL)	122 ± 9	124 ± 13	0.991
	Triglyceride (mg/dL)	117 ± 48	112 ± 67	0.343
	Fasting blood glucose (mg/dL)	96 ± 14	100 ± 18	0.235

BP, blood pressure.

In addition, there were no significant differences in the parameters associated with LV structure and function, including LVEF, between the two groups (Table 2). No participant showed impaired LV wall motion. Doppler measurements showed that the velocities of mitral valve inflow and tissue Doppler velocity of the mitral annulus did not differ between the two groups. Although mild mitral regurgitation was observed in one participant and a bicuspid aortic valve was seen in another participant in the flight group, no other severe valve-related problems were observed. Among the parameters associated with the right heart, there were no differences in TR peak velocity, RVSP, TAPSE, IVC diameter, or IVC collapsibility between the two groups.

**Table 2:** Comparison of echocardiographic parameters between the non-flight and flight groups.

Echocardiographic parameters	Non-flight group (n = 16)	Flight group (n = 29)	*P* value
LVID diastole (mm)	50 ± 4	52 ± 5	0.250
LVID systole (mm)	33 ± 4	33 ± 4	0.948
IVS diastole (mm)	8 ± 2	9 ± 1	0.093
IVS systole (mm)	13 ± 2	14 ± 2	0.126
LVPW diastole (mm)	8 ± 1	9 ± 1	0.053
LVPW systole (mm)	13 ± 2	14 ± 2	0.148
Aorta (mm)	28 ± 5	28 ± 4	0.887
LA (mm)	34 ± 4	36 ± 5	0.221
LVEF (%)	65 ± 6	66 ± 7	0.455
RVOT peak velocity (m/s)	0.8 ± 0.1	0.8 ± 0.1	0.532
LVOT peak velocity (m/s)	1.3 ± 0.2	1.2 ± 0.2	0.255
TR peak velocity (m/s)	1.7 ± 0.5	1.8 ± 0.4	0.294
RVSP (mmHg)	17 ± 6	19 ± 4	0.405
E velocity (m/s)	0.7 ± 0.1	0.7 ± 0.3	0.353
A velocity (m/s)	0.6 ± 0.1	0.5 ± 0.1	0.516
e′ velocity (m/s)	0.10 ± 0.03	0.10 ± 0.03	0.459
TAPSE (cm)	2.3 ± 0.2	2.4 ± 0.2	0.209
IVC diameter expiration (cm)	1.3 ± 0.5	1.5 ± 0.4	0.095
IVC diameter Inspiration (cm)	0.8 ± 0.2	1.0 ± 0.4	0.105
IVC collapsibility (%)	40 ± 19	38 ± 14	0.522
Valve abnormality (n (%))	0 (0%)	2 (7%)	0.531
RWMA (n (%))	0 (0%)	0 (0%)	–

LVID, left ventricle internal diameter; IVS, interventricular septum; LVPW, left ventricular posterior wall; LA, left atrium; LVEF, left ventricular ejection fraction; RVOT, right ventricle outflow tract; LVOT, left ventricle outflow tract; TR, tricuspid regurgitation; RVSP, right ventricular systolic pressure; E, early diastolic mitral inflow; A, late diastolic mitral inflow; e′, early diastolic mitral annulus; TAPSE, tricuspid annular plane systolic excursion; IVC, inferior vena cava; RWMA, regional wall motion abnormality.

When corrected for covariables in the multiple regression analysis performed in the flight group, TAPSE showed a positive correlation with flight time and negative correlation with pilot height (Table 3).

**Table 3:** Multivariate linear regression analysis for TAPSE.

Variable	Coefficient	Standard error	*P* value
Total flight time (h)	0.070	0.021	0.042
Age (years)	− 0.100	0.044	0.106
Height (m)	− 8.461	2.536	0.045

Adjustment for age, total flight time, height, weight, systolic BP, total cholesterol, LDL, LVID, IVSD, LVPW, LA diameter, LVEF, RVSP, E velocity, TR peak velocity, and IVC collapsibility.

TAPSE, tricuspid annular plane systolic excursion; BP, blood pressure; LDL, low-density lipoprotein; LVID, left ventricle internal diameter; IVSD, interventricular septum diastole; LVPW, left ventricular posterior wall; LA, left atrium; LVEF, left ventricular ejection fraction; RVSP, right ventricular systolic pressure; E, early diastolic mitral inflow; TR, tricuspid regurgitation; IVC, inferior vena cava.

## Discussion

In the present study, the flight group showed no changes in cardiac function and structure based on echocardiographic parameters compared with those in the non-flight group. All the participants had normal echocardiographic parameters, LV wall motion, and valve function. Multivariate regression analysis was performed on data from the fighter pilots, using flight time as a continuous variable, to evaluate the accumulation of G-forces. The results showed that after adjusting for various confounding variables, flight time might be associated with parameters related to right heart function, including TAPSE.

In a previous study of short-term follow-up echocardiography before and after flight in jet pilots, no differences in cardiac- and aorta-related parameters were observed using 2D and M-mode images^18^. When the same research group conducted a prospective study on jet pilots over a 10-year period, they did not find any significant differences in cardiac function^19^. Furthermore, our study showed no significant differences in the cardiac chamber size, LVEF, or Doppler findings between the flight and non-flight groups, suggesting that flight experience had no significant effect on the risk of structural cardiac changes and LV function. Unlike in previous studies, right heart parameters, such as TR velocity, RVSP, IVC collapsibility, and TAPSE, were included in this study; however, there was no difference between the two groups with respect to these parameters. Thus, when the participants were simply divided based on flight experience, there was no significant difference in the left and right cardiac function between the flight and non-flight groups. However, the comparative analysis between these two groups was limited because the cumulative effect of G acceleration on the heart could not be estimated. Separately, there was a difference in weight and cholesterol levels between the non-flight and flight groups, and both were higher in the flight group. There is no clear evidence that flight experience increases weight or cholesterol, but we suspect that there may be an effect of age^20,21^, as the flight group was slightly older and empirically more experienced, although it was not statistically significant in our sample.

The strength of this study is that total flight time was used as a quantitative independent variable. In the multivariate linear regression analysis for parameters associated with cardiac function, total flight time had a significant effect on TAPSE, after adjusting for other covariates. TAPSE is primarily considered as an index of RV systolic function; however, some studies have shown that it correlates with RV filling pressure, which is a measure of RV diastolic function^17,22^. In this study, TAPSE, an index of systolic and diastolic functions of the RV, showed a positive correlation with total flight time, suggesting that flight experience might affect right heart function. Although the statistical power of this association is small, it is worth considering given the pilots’ repetitive exposure to high G-acceleration over a long period and the gradual physiological changes caused by anti-G maneuvers. The hypothesis that a high-G acceleration flight may affect right heart function has been proposed in several previous studies. In this regard, a study comparing the echocardiographic results of general and jet pilots suggested that the RV size was more extensive in jet pilots^23^. Furthermore, a similar study comparing the echocardiographic results of jet fighters and general pilots revealed differences in parameters related to RV function, such as peak tricuspid valve velocity and mean pulmonary artery pressure^24^. Another study comparing echocardiographic results between pilots and controls showed that pilots had higher rates of pulmonary valve insufficiency and TR than those in controls^25^. The authors explained these results as secondary changes in RV pressure caused by continuous anti-G maneuvers to prevent loss of consciousness due to gravity (G-LOC) at high-G acceleration.

The anti-G maneuver is a type of positive-pressure ventilation that prevents G-LOC by reducing venous return. During strain, venous return to the heart decreases, and right heart capacity and blood volume passing through the right heart may decrease^12,26,27^. Right heart pressure may be affected by increased intraabdominal pressure^28,29^. In the relaxation phase, venous return and diastolic filling recover and pressure in the right heart normalizes^26^. Positive-pressure breathing could result in decreased RV filling owing to a decrease in the pressure gradient of the systemic venous return, similar to the Valsalva maneuver^30–32^. Repeated execution and interruption of positive-pressure ventilation may alter right heart function and pressure. That is, breathing training in these special fighter pilots may provide clues to the effects on right heart function.

In contrast, pilot height was negatively correlated with TAPSE. One proposed explanation for this relationship is that taller individuals may have a larger thoracic cavity, which may result in a greater mechanical disadvantage and lead to increased stretching of the RV myocardium. This can impair RV function and decrease TAPSE^33^. However, the relationship between height and TAPSE is not yet well established, and further research is needed to confirm this association and to better understand its underlying mechanisms.

The small sample size and unified sex of the study participants may have limited the generalizability of the findings. In addition, most pilots had varying levels of exposure to different types of jet planes over time, and it was difficult to determine whether all experiences of high-G acceleration could be considered uniform. Therefore, caution must be exercised when interpreting these results and generalizing the effects of high-G acceleration on cardiac function. Future studies with more standardized G-exposure protocols and larger sample sizes are necessary to further explore this relationship.

In conclusion, fighter pilots’ repeated exposure to high-G acceleration forces and anti-G maneuvers did not cause changes in cardiac structure; however, the exposure may be associated with changes in right heart function. Therefore, echocardiographic assessment, including that of right heart function and general cardiac parameters, may be beneficial for the cardiovascular health of jet pilots.

## Methods

### Study population

**Figure 1:**
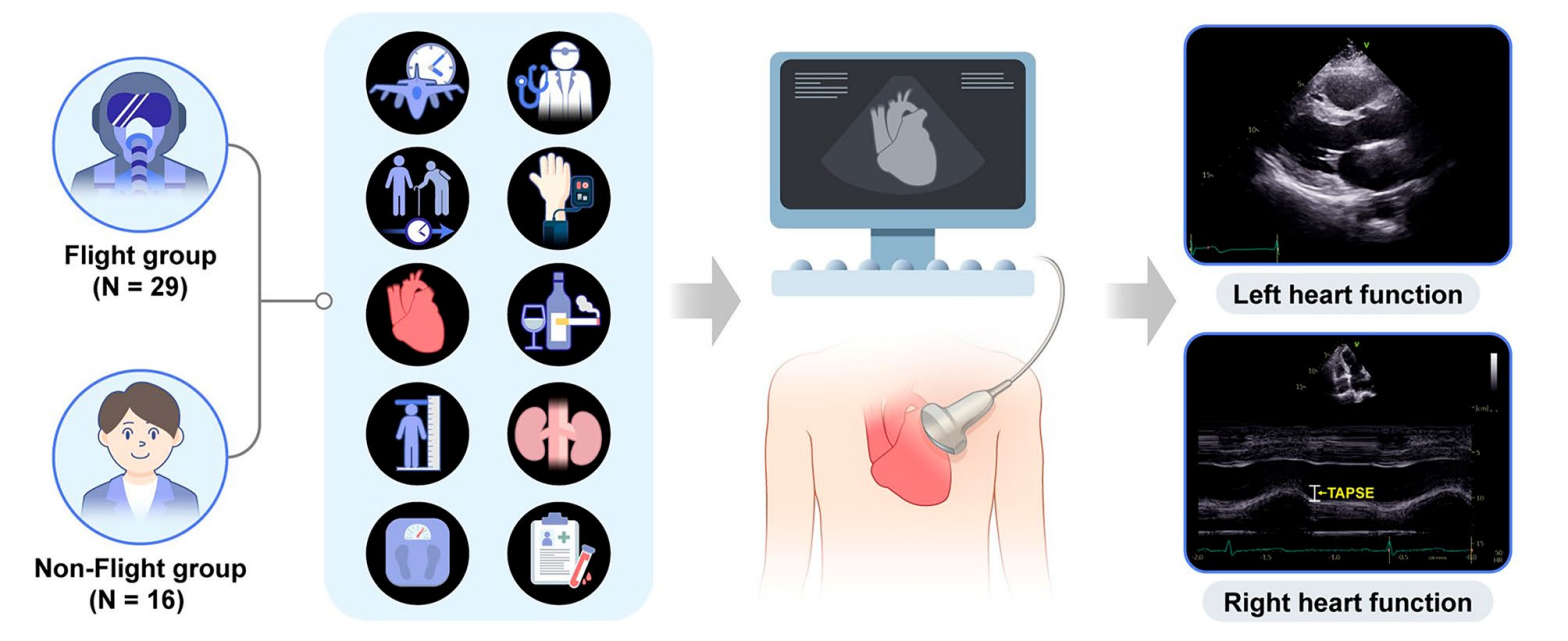
Study population and design. TAPSE, tricuspid annular plane systolic excursion.

To assess the influence of G acceleration on the heart, the participants were divided into two groups: flight and non-flight. Korean air force fighter pilots who experienced “air-to-air” missions and high-G acceleration flights of more than 6 G between July 2019 and June 2020 were included in the flight group. Non-flying trainees, including Air Force Academy students and pre-pilots, were recruited as controls in the non-flight group (Fig. 1).

Regular health check-up data included age, height, weight, blood pressure (BP), blood total cholesterol, low-density lipoprotein (LDL), high-density lipoprotein (HDL), triglycerides, and fasting blood glucose levels. In addition, data, including total flight time, medical history, family history, alcohol intake, and smoking status, were collected from the pilots’ flight records and questionnaires. This study was supported by the Korean Air Force Aerospace Medicine Research Center and informed consent was waived by the Korean Air Force Aerospace Medicine Research Center Institutional review board (IRB)/ethics committee due to the anonymity and retrospective nature of the study (ASMC-20-IRB-002). Participants in this study were conducted in accordance with appropriate guidelines and the Declaration of Helsinki.

### Echocardiographic data

**Figure 2:**
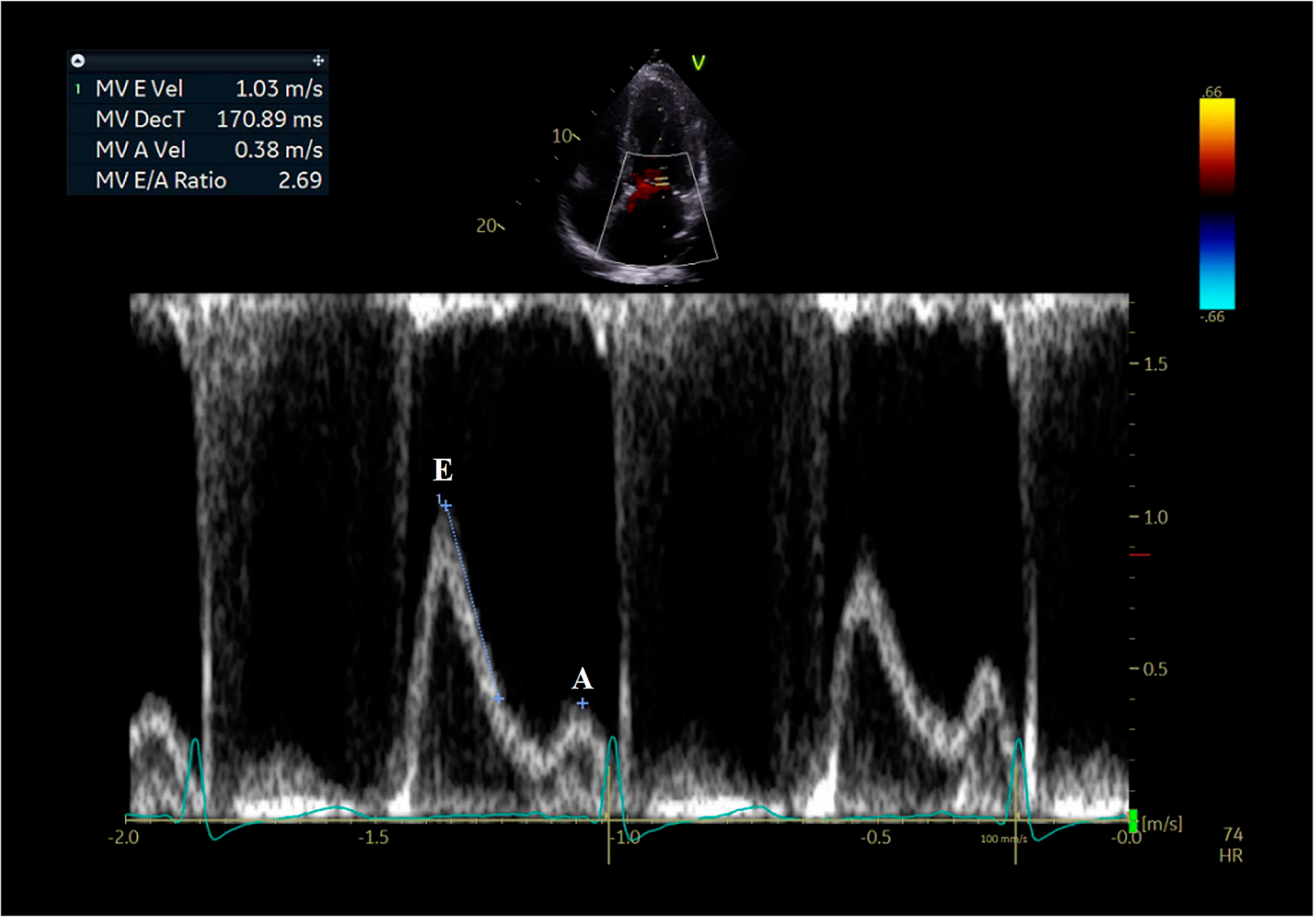
Measurement of early (E) and late (A) diastolic peak velocities. MV, mitral valve; Vel, velocity; DecT, deceleration time.

Echocardiographic data included the diameters of the left ventricle (LV), interventricular septum (IVS), LV posterior wall (PW), left atrium (LA), and aorta on two-dimensional (2D) images. LV ejection fraction (EF) was calculated using the modified Simpson’s method^13^. LV diastolic function was assessed by measuring early (E) and late (A) diastolic peak velocities acquisitioned by the pulsed-wave Doppler in apical four-chamber view with color flow (Fig. 2). Doppler sample volume was obtained at mitral leaflet tips, and the values of E and A diastolic peak velocity were obtained from the peak modal velocities of early diastole and late diastole at the leading edge of the spectral waveform of the pulsed-wave Doppler. Septal peak early diastolic mitral annular velocity (e′) was measured by peak modal velocity in early diastole at the leading edge of spectral waveform, using tissue Doppler imaging at septal basal regions in the apical four-chamber view^14^. Valve abnormalities were defined as grade ≥ 2 valvular regurgitation or significant anatomical abnormalities. Pulsed-wave Doppler recordings were obtained from the parasternal short-axis view and apical three- or five-chamber views to estimate the velocity of the LV and right ventricular outflow tract^15^.

Right ventricular function was evaluated by estimating the tricuspid annular plane systolic excursion (TAPSE) using anatomical M-mode imaging of the focused apical four-chamber view of the right ventricle (RV). The diameter of the inferior vena cava (IVC) was measured in the subcostal view and IVC collapsibility was calculated as the ratio of IVC diameter reduction during inspiration to that during expiration. The pressure gradient (P) is determined by the velocity in the orifice (V), which can be described by the Simplified Bernoulli equation [ΔP = 4V^2^]^16^. Right ventricular systolic pressure (RVSP) was determined from peak tricuspid regurgitant jet velocity (TR Vpeak), using the simplified Bernoulli equation and combining this value with an estimate of the right atrium (RA) pressure estimated from IVC diameter and respiratory changes in the subcostal view [RVSP = 4(TR Vpeak)^2^ + RA pressure]^17^.

All echocardiographic studies were performed at the Republic of Korea Air Force Aeromedical Center using the same device (Philips iE33; Philips Medical Systems, Andover, MA, USA). All studies were performed and interpreted by one of the two experienced cardiologists specializing in echocardiography and aerospace cardiology. All the examinations were performed by an experienced cardiologist. Echocardiographic parameters were measured according to the American Society of Echocardiography guidelines and standard committee recommendations for echocardiographic measurements.

### Statistical analysis

Statistical analyses were performed using Statistical Package for the Social Sciences Statistics for Windows (version 18.0; SPSS Inc., Armonk, NY, USA). Continuous variables were compared between the flight and non-flight groups using the Student’s t-test for parametric analysis and the Mann–Whitney U test for non-parametric analysis. Chi-squared and the Fisher’s exact tests were used to compare categorical variables between the two groups. Statistical significance was set at *P* < 0.05. Furthermore, a multivariate linear regression analysis was performed to determine whether total flight time, as a continuous variable, affected the parameters associated with cardiac function in fighter pilots. In the multivariate linear regression analysis, age, total flight time, height, weight, systolic BP, total cholesterol, LDL, LV diameter, IVS diameter, LVPW diameter, LA diameter, LVEF, RVSP, early diastolic mitral inflow velocity, TR peak velocity, and IVC collapsibility were adjusted.

### Ethics approval

This study was approved by the Institutional Review Board of the Korean Air Force Aerospace Medicine Research Center (ASMC-20-IRB-002).

### Consent to participate

The requirement for informed consent to participate in the study was waived.

## Data Availability

Data supporting the findings of this study are available from the corresponding author upon reasonable request and with permission from the medical research centers where the authors collected the data retrospectively.
